# ER stress and unfolded protein response in amyotrophic lateral sclerosis—a controversial role of protein disulphide isomerase

**DOI:** 10.3389/fncel.2014.00402

**Published:** 2014-12-02

**Authors:** Merja Jaronen, Gundars Goldsteins, Jari Koistinaho

**Affiliations:** ^1^Department of Neurobiology, A. I. Virtanen Institute for Molecular Sciences, University of Eastern FinlandKuopio, Finland; ^2^Center for Neurologic Diseases, Brigham and Women’s Hospital, Harvard Medical SchoolBoston, MA, USA

**Keywords:** ALS, ER stress, oxidative stress, neurodegeneration, motoneuron, glia

## Abstract

Accumulation of proteins in aberrant conformation occurs in many neurodegenerative diseases. Furthermore, dysfunctions in protein handling in endoplasmic reticulum (ER) and the following ER stress have been implicated in a vast number of diseases, such as amyotrophic lateral sclerosis (ALS). During excessive ER stress unfolded protein response (UPR) is activated to return ER to its normal physiological balance. The exact mechanisms of protein misfolding, accumulation and the following ER stress, which could lead to neurodegeneration, and the question whether UPR is a beneficial compensatory mechanism slowing down the neurodegenerative processes, are of interest. Protein disulphide isomerase (PDI) is a disulphide bond-modulating ER chaperone, which can also facilitate the ER-associated degradation (ERAD) of misfolded proteins. In this review we discuss the recent findings of ER stress, UPR and especially the role of PDI in ALS.

## Introduction

Intracellular protein aggregates are characteristic of neurodegenerative diseases, including amyotrophic lateral sclerosis (ALS; Ross and Poirier, 2004), the most common motoneuron disease characterized by a selective death of upper and lower motoneurons. The exact role of protein aggregates in disease pathology is still under debate (Ross and Poirier, 2005). In ALS the majority of aggregates are ubiquitinated inclusions (Wood et al., 2003) typically containing trans-activation response element (TAR) DNA binding protein 43 (TDP-43; Neumann et al., 2006) and also mutant Cu, Zn-superoxide dismutase (SOD1) in SOD1-linked familial ALS (Shibata et al., 1996) and mutant SOD1 mouse (Watanabe et al., 2001) and cell culture models (Lee et al., 2002). A likely underlying cause for protein accumulation is oxidative stress causing dissociation of SOD1 dimers to monomers and subsequently leading to aggregation (Rakhit et al., 2004). This can then together with malfunctioning proteasome degradation machinery contribute to the motoneuron dysfunction (Marino et al., 2014). Mutant SOD1s have also an ability to catalyze aberrant oxidative reactions which damages SOD1 itself (Andrus et al., 1998) possibly resulting in aggregation of mutant SOD1 (Valentine and Hart, 2003). Recent studies have further highlighted the importance of protein aggregation, especially in the case of TDP-43, in ALS pathology, as they have demonstrated that the induction of autophagy can enhance the TDP-43 turnover and neuronal survival (Barmada et al., 2014).

## ER stress and unfolded protein response in ALS

As the first compartment in the secretory pathway, endoplasmic reticulum (ER) is responsible for protein synthesis, posttranslational processing, folding of newly synthesized proteins, and finally delivering the biologically active proteins to their proper target sites. The rate-limiting step in the secretory pathway is the transit to Golgi complex. If ER capacity is transcended and influx to the ER is excessive, the normal physiological state of the ER is disrupted leading to ER stress. There turn to its normal physiological balance then requires activation of the unfolded protein response (UPR) signaling pathway. If the UPR fails to restore the cell integrity, cell death signaling cascades are activated and the cell undergoes apoptosis (Schröder and Kaufman, 2005).

Changes in ER morphology have been observed in ALS patients and the G93A-SOD1 mice (Lautenschlaeger et al., 2012). G93A-SOD1 mice exhibit dilated rough ER (rER) accompanied with ribosomal detachment at preclinical and early symptomatic stages (Dal Canto and Gurney, 1995). Similar findings were demonstrated in postmortem samples of sporadic ALS (sALS) patients as researchers reported fragmentation of the rER, irregular distension of the rER cisternae and a detachment of ribosomes in degenerating anterior horn cells (Oyanagi et al., 2008). A recent study demonstrated deposits of granular or amorphous material in the ER lumen of sALS patients, indicating accumulation of misfolded proteins which could then in turn cause ER stress (Sasaki, 2010). In addition, Golgi apparatus become fragmented both in ALS patients (Fujita et al., 2000) and in G93A-SOD1 mice (Stieber et al., 2000). Although the results indicate an early interference in the ER balance, the manifold disease course and specific disease stage of an individual cell make it difficult to draw an accurate picture of the ongoing process (Lautenschlaeger et al., 2012).

## Causes of ER stress

Accumulation of SOD1 has been considered one of the reasons for ER stress in ALS, as mutant SOD1 colocalizes with ER markers, including glucose-related protein 78 (Grp78) and calnexin (Wate et al., 2005; Kikuchi et al., 2006). Further evidence for linking UPR to SOD1 accumulation was gained by a discovery that protein disulphide isomerase (PDI), an ER chaperone, is up-regulated both in ALS patients and G93A-SOD1 mice (Atkin et al., 2006) co-localizing with accumulated mutant SOD1 (Atkin et al., 2006). Mutant SOD1 may interact with Derlin-1 and cause dysregulation of ER-associated degradation (ERAD), thereby leading to activation of ER stress-induced apoptosis signal-regulating kinase 1 (ASK1) and apoptosis (Nishitoh et al., 2008).

Another possible cause for ER stress in ALS is the imbalance of ER calcium homeostasis (Grosskreutz et al., 2010) as protein processing and folding are calcium dependent (Kuznetsov et al., 1992). Importantly, decreased ER calcium content contributes to ER stress in ALS (Jaiswal and Keller, 2009). The ER mitochondria calcium cycle hypothesis proposes that the increased calcium release from ER is coupled with calcium uptake by mitochondria and that calcium is then transported back to ER (Grosskreutz et al., 2010). This hypothesis could explain why dysfunction in the mitochondrial calcium storage (Damiano et al., 2006) would lead to disruption of the ER refilling and subsequently to ER stress and UPR (Grosskreutz et al., 2010).

## Unfolded protein response in ALS

Three major ER stress sensors detect accumulation of unfolded proteins: double-stranded RNA-activated protein kinase (PKR)-like ER kinase (PERK), inositol requiring enzyme 1 (IRE1), and activating transcription factor 6 (ATF6; Schröder and Kaufman, 2005). Inositol required enzyme 1 and PERK, are type I transmembrane proteins with protein kinase activity (Liu et al., 2002) while ATF6 is a type II transmembrane protein, whose cytosolic domain can translocate to the nucleus and activate UPR relevant genes (Haze et al., 1999). While the ER luminal domains of all three ER stress sensors normally bind to the ER chaperone GRP78, under ER stress GRP78 dissociates from these sensors to bind to the misfolded proteins and enables the activation of UPR sensors (Kaufman, 1999). As stated before, mutant SOD1 has been demonstrated to bind to and co-localize with GRP78, which increases its expression in ALS mice prior to the motor symptoms (Tobisawa et al., 2003).

### The double-stranded RNA-activated PERK

Dimerization and trans-autophosphorylation result in activation of the PERK kinase domain (Harding et al., 1999). Activation of PERK in turn leads to phosphorylation of the eukaryotic initiation factor-2 (eIF2α) inhibiting general translation initiation and protecting ER against an overload of newly synthesized proteins (Harding et al., 1999). Paradoxically eIF2α phosphorylation increases translation of activating transcription factor 4 (Lu et al., 2004). ER kinase has also another substrate, nuclear factor erythroid 2-related factor 2 (Nrf2; Cullinan and Diehl, 2006). ER kinase pathway has been implicated in sALS (Hetz et al., 2009). Moreover, increased amounts of phospho-PERK-PERK (Atkin et al., 2006, 2008; Saxena et al., 2009) and phospho-eIF2α (Saxena et al., 2009) both in G93A-SOD1 mice and Neuro2a cells transfected with mutant SOD1 have been reported.

### Inositol requiring enzyme 1 (IRE1)

Endoplasmic reticulum stress elicits autophosphorylation of IRE1 inducing its RNAse activity (Liu et al., 2002). IRE1 mediated endoribonuclease activity consequently leads to non-conventional splicing of XBP1 (X-box binding protein 1; Calfon et al., 2002). Spliced XBP1 in turn translocates to the nucleus and controls genes related to protein quality control, protein folding, components of the ERAD pathway and genes required for lipid synthesis (Sriburi et al., 2004). Postmortem spinal cord samples from ALS and ALS mice manifest increased amounts of IRE1. Interestingly, ALS mice had augmented IRE1 amounts before the onset of symptoms (Atkin et al., 2006, 2008). Further studies have revealed up-regulation of phosphorylated IRE1 and increased amount of spliced XBP1 in G93A-SOD1 mice (Kikuchi et al., 2006). Accordingly, studies with Neuro2a cells expressing G85R-SOD1 demonstrated increased splicing and nuclear translocation of XBP1 mRNA (Oh et al., 2008). The unphosphorylated and unspliced forms of IRE1 and XBP1 levels are not changed indicating that these forms could operate as an activable pool (Lautenschlaeger et al., 2012). Surprisingly, knocking down IRE1 and XBP1 by shRNA in NSC-34 cells transfected with SOD1 mutant caused decreased SOD1 aggregation and improved cell survival (Hetz et al., 2009). Augmented autophagy has been hypothesized to be the reason for the protective outcome. Generation of a knockout/transgenic mouse line by crossbreeding G86R-SOD1 and XBP1 Nes−/− (Hetz et al., 2008) demonstrated several autophagic signs further strengthening the autophagy hypothesis. XBP1 Nes−/−—G86R-SOD1 mice had a slightly prolonged life span in females whereas males showed no improvement (Hetz et al., 2009).

### The activating transcription factor 6 (ATF6)

Endoplasmic reticulum stress translocates ATF6 to Golgi apparatus where it is cleaved by two proteases (Haze et al., 1999). Following cleavage, the cytosolic domain of ATF6 is translocated to nucleus where it activates UPR-related genes (Gotoh et al., 2002; Yoshida et al., 2003) Elevated levels of ATF6 have been reported in ALS patients and G93A-SOD1 mice (Atkin et al., 2006, 2008). In addition, cleavage and translocation of ATF6 in Neuro2a cells transfected with mutant SOD1 has been verified (Oh et al., 2008) and knocking down ATF6 in NSC-34 cells transfected with mutant SOD1 was found to increase SOD1 aggregation (Hetz et al., 2008). Importantly, mutations in vesicle-associated membrane protein-associated protein B (VAPB), which have been connected with late-onset motoneuron disease, associates with intracellular membranes (Nishimura et al., 2004) as well as with UPR (Gkogkas et al., 2008). Both native and mutant VABP interact with ATF6 and reduce its capability to promote transcription of XBP1. The mutant VAPB is a much more potent inhibitor of ATF6 than the wild type VAPB, which may contribute to the pathological mechanisms of ALS (Gkogkas et al., 2008).

## Controversial role of PDI in ALS

As seen above, the majority of studies related to UPR in ALS have concentrated on motoneuronal UPR. However, damaged white matter has been reported in several neurological disorders (Matute, 2006) and, interestingly, loss of large myelinated fibers in the corticospinal tracts and ventral roots has been demonstrated in ALS patients (Underwood et al., 2011). Importantly, motoneurons that develop ER stress response are coupled with microglial activation and consequent axonal degeneration (Saxena et al., 2009). Moreover, up-regulation of UPR markers PDI and GADD34 have been demonstrated in glial cells in the spinal cord of G93A-SOD1 mice right after disease onset and shown segregation of UPR into ventral horn astrocytes and white matter microglia (Jaronen et al., 2013). This segregation is likely to reflect variable roles of UPR in astrocytes around degenerating motoneuron cell bodies and microglia around both motoneuron cell bodies and neurites. In view of the early degenerative changes in motoneuron axons and the role of microglia in front line defense, it is not surprising that microglial UPR precedes and/or dominates over astrocytic UPR during early motoneuron degeneration.

### PDI inactivation may contribute to protein aggregation

**Figure 1:**
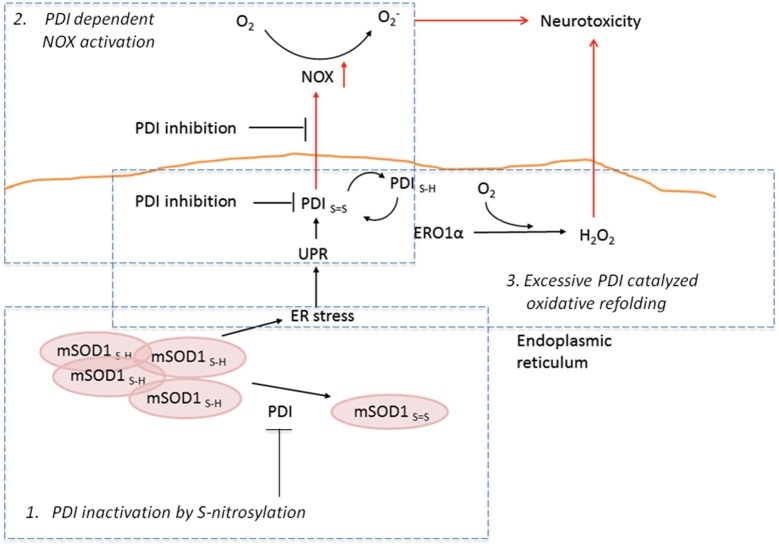
**The role of PDI in ALS**. At the early stages of ALS, PDI can prevent SOD1 aggregation by re-assembling SOD1 to its active form. Protein disulphide isomerase inactivation by S-nitrosylation increases ER stress (1). Further on in the disease, the aggregation of SOD1 can lead to ER stress following UPR. Interestingly, augmented UPR can result in increased PDI activation. This can in turn result in NOX activation, leading to increased superoxide production and finally neurotoxicity (2). The second possible route for PDI-related neurotoxicity is through ERO1α; PDI activity will activate ERO1α, which will then in turn produce hydroperoxide and result in neurotoxicity (3).

Protein disulphide isomerase is an enzyme of a thioredoxin superfamily primarily functioning in the ER as a chaperone protein. It facilitates the rearrangements of disulphide bonds via catalysis of thiol-disulphide exchange (Wilkinson and Gilbert, 2004; Ellgaard and Ruddock, 2005). In addition to its well-known role in the ER, PDI has been found in other cellular localizations, such as cytosol and mitochondria, where its physiological role is not yet completely clear (Rigobello et al., 2001; Turano et al., 2002; Wilkinson and Gilbert, 2004). However, the mitochondrion-associated PDI can induce apoptosis through mitochondrial outer membrane permeabilization when accumulating at high levels in response to misfolded proteins (Hoffstrom et al., 2010). Up-regulation of PDI has been demonstrated in ALS (Atkin et al., 2006, 2008). In G93A-SOD1 rats PDI expression is increased early in the disease progression declining sharply towards the end stage (Ahtoniemi et al., 2008). The up-regulation of PDI in the early symptomatic stages of ALS (Ahtoniemi et al., 2008) might be due to the attempt to resolve the misfolding and aggregating SOD1, but as the oxidative damage increases (Goldsteins et al., 2008) PDI becomes oxidized and loses its ability to function as a disulphide bond-rearranging enzyme (Figure 1). Recent report has demonstrated that S-nitrosylated and inactivated PDI can increase mutant SOD1 aggregation and trigger neuronal cell death (Jeon et al., 2014).

### PDI upregulation may cause NOX activation

Regardless of the fact that PDI is generally thought to act as a compensatory survival supporting enzyme, the other side of the PDI coin might not be as beneficial as once assumed. Recent studies have shown that in non-neuronal cells, such as vascular cells and peripheral macrophages, PDI is associated with NADPH oxidase (NOX) and act as a redox-sensitive regulatory protein of several NOX isoforms (Janiszewski et al., 2005; Laurindo et al., 2008; Santos et al., 2009a). NADPH oxidase is an enzyme that generates superoxide by transferring electrons from NADPH inside the cell across the membrane and coupling these to molecular oxygen to produce superoxide anion, a reactive free-radical. Indeed, *in vivo* ALS studies indicate that NOX activation and superoxide production are elevated in microglia and may contribute to motoneuron death (Wu et al., 2006). Furthermore, ER stress capable of inducing UPR has been previously shown to result in NOX activation, leading to increased superoxide production in peripheral macrophages (Li et al., 2010). As UPR has been demonstrated in microglia in the spinal cords of G93A-SOD1 mice (Jaronen et al., 2013), we hypothesize that PDI activity might be coupled to NOX-mediated reactive oxygen species (ROS) production during UPR. The view is supported by the finding that induction of UPR results in NOX activation and this activation is PDI-dependent (Jaronen et al., 2013). Moreover, siRNA-mediated down-regulation of PDI expression was found to reduce NOX activation. Similar results were obtained when human primary monocytes, rat primary microglia and murine macrophage type of cells were used. These *in vitro* data suggest that PDI is a significant regulator of UPR-induced NOX activation in cells of hematopoietic origin. Transient expression of G93A-SOD1 inflicted augmented NOX activation in microglia BV-2 cells suggesting that mutant SOD1 is capable of triggering the UPR and finally superoxide production (Figure 1). Although several models have been proposed where PDI interacts with catalytical or regulatory subunits of NOX (Laurindo et al., 2008), the exact mechanism of how PDI activates NOX remains unclear. Based on the current knowledge PDI reductase activity may be required as bacitracin, an inhibitor of PDI reductase activity (Dickerhof et al., 2011), is able to suppress superoxide production in several cell types.

### Excessive PDI catalyzed refolding may contribute to oxidative stress

The main site of PDI function is the ER, where the redox conditions are very different from cytosol, enabling the protein folding. Glutathione is one of the key players in controlling the redox status of ER as it has been shown that glutathione can provide oxidizing equivalents for disulphide formation (Hwang et al., 1992). However, oxidoreductin Ero1 is thought to act as a primary electron acceptor in the disulphide bond formation, transferring oxidizing equivalent to its substrate PDI (Sevier et al., 2007). Ero1 oxidizes the active cysteinyl thiol groups in PDI, enabling it then in turn to oxidize the client protein and create a disulphide bond. As Ero1 acts as an acceptor of electrons from PDI, it passes the electrons to molecular oxygen creating harmful hydroperoxide (Figure 1; Higa and Chevet, 2012). Furthermore, reduced glutathione may be necessary for isomerization of improper disulphide bonds, resulting in oxidized glutathione (Margittai and Bánhegyi, 2010). These hydroperoxide and oxidized glutathione byproducts are thought to be dangerous (Tu and Weissman, 2002; Margittai and Bánhegyi, 2010) and form a link between ER stress and oxidative stress (Harding et al., 2003; Haynes et al., 2004; Malhotra et al., 2008). However, no clear consensus on whether the Ero1-mediated extensive oxidation in the ER leads to augmented oxidative stress or acts as a part of homeostatic redox control mechanisms, has been reached (Appenzeller-Herzog, 2011). Interestingly, a recent study by Shepherd et al. (2014) shed more light over the companionship of PDI and Ero1, demonstrating that PDI has ability to catalyze both the activation and inactivation of its own catalyst Ero1.

## Conclusion

Endoplasmic reticulum stress is a characteristic of neurodegenerative diseases, including ALS. While UPR is thought to be an adaptive and protective reaction of cells to overwhelming ER stress, the cellular response triggered by protein aggregation and UPR together may lead to misbalance in protein folding pathway and result in increased ROS production. The following increased oxidative stress upon UPR can be regarded as a union of a number of both proapoptotic and proadaptive mechanisms (Santos et al., 2009b), increased PDI expression being an integral part of the latter. However, keeping in mind that high levels of PDI in response to misfolded proteins, is also capable of promoting a cell death cascade (Hoffstrom et al., 2010), the control of PDI expression offers an interesting therapeutic strategy. In microglia cells ROS production may indeed depend on PDI, which associates with NOX and regulates its function. In agreement with our findings, recent studies have shown that overexpression of PDI promotes NOX activation in vascular smooth muscle cells (Fernandes et al., 2009). Furthermore, our notion is also supported by findings that PDI closely associates with p22phox subunit of phagocyte NOX, and that NOX activation directly correlates with PDI expression levels (Santos et al., 2009a). Currently the main scope for the role of PDI in protein aggregation linked neurodegeneration has been focused at its function in maintenance of native protein structure in neurons. Nevertheless upon excessive protein misfolding the penalty of oxidative stress originating from oxidative folding may exceed ER adaptive capabilities in neuronal cells and cause aberrant NOX activation in microglia.

## Conflict of interest statement

The authors declare that the research was conducted in the absence of any commercial or financial relationships that could be construed as a potential conflict of interest.
